# The air pollutant phenanthrene disrupts calcium cycling and alters repolarization in female sheep ventricular cardiomyocytes

**DOI:** 10.14814/phy2.70471

**Published:** 2025-08-26

**Authors:** C. R. Marris, S. N. Kompella, A. W. Trafford, J. C. Hancox, H. A. Shiels

**Affiliations:** ^1^ Division of Cardiovascular Sciences, Faculty of Biology Medicine and Health University of Manchester Manchester UK; ^2^ Cardiovascular Research Laboratories, Biomedical Sciences Building Bristol Medical School, University of Bristol Bristol UK; ^3^ Present address: Dementia Research Institute, School of Medicine Cardiff University Cardiff UK

**Keywords:** action potential, Ca^2+^ transient, I_K_, large animal model, ovine, tricyclic polyaromatic hydrocarbons

## Abstract

Multiple epidemiological studies link cardiac dysfunction with increased levels of air pollution. While cellular mechanisms underlying such dysfunction are yet to be fully elucidated, a proposed mediator of this effect is phenanthrene, a 3‐ringed polycyclic aromatic hydrocarbon (PAH). Here, we used ventricular myocytes freshly isolated from healthy female sheep (*Ovis aries*) to study the impact of acute phenanthrene exposure on cardiac electrophysiology and intracellular Ca^2+^ cycling in a large mammalian model. We observed ~37% (*n* = 11) shortening of the action potential duration (APD) at 90% repolarization following exposure to 25 μM phenanthrene. This APD shortening occurred despite a concentration‐dependent inhibition of the delayed rectifier potassium current (I_Kr_), with maximal inhibition of ~56% with 25 μM phenanthrene. Phenanthrene also reduced the calcium transient amplitude by ~40% (*n* = 10), slowed the rate of calcium transient decay by ~60% (*n* = 10), but had no effect on the peak current amplitude of I_Ca_. Notably, preincubation of ventricular myocytes with pharmacological agents that inhibit sarcoplasmic reticulum calcium cycling by inhibiting SERCA activity and ryanodine receptor function prevented phenanthrene‐dependent changes in the calcium transient amplitude and time‐course. Together, these data show acute exposure to phenanthrene impairs electrical activity and intracellular calcium cycling in ventricular myocytes of healthy sheep.

## 1 INTRODUCTION

Air pollution is a significant global health concern, contributing to millions of premature deaths each year. Recent estimates indicate that in 2021, air pollution was responsible for approximately 8.1 million deaths worldwide, making it the second leading risk factor for death globally (UNICEF, 2024). The harmful effects of air pollution on cardiovascular health are well documented (Franklin et al., 2015). Epidemiology has revealed strong correlations between air pollution levels and an elevated risk of cardiovascular disease (CVD) (Khoshakhlagh et al., 2024; Mumtaz et al., 2021; Rhee et al., 2024; Sigsgaard & Hoffmann, 2024; Zhang et al., 2023), including cardiac‐related hospitalizations during acute spikes in poor air quality (Yu et al., 2024). In England, it is estimated that 8400 heart and circulatory deaths per year are attributable to particulate matter (PM) pollution (Hellgren et al., 2021). Without intervention, deaths from outdoor air pollution are projected to double by 2050 (Lelieveld et al., 2023).

Polycyclic aromatic hydrocarbons (PAHs) are a family of ubiquitous pollutants present in air, soil, and water. The cardiotoxicity of the low‐molecular weight PAHs (containing 3 or less benzene rings) came to prominence following the syndrome of defects noticed in fishes exposed to the 1989 Exxon Valdez oil spill and 2010 Deepwater Horizon wellhead blowout (Carls et al., 2008; Incardona et al., 2004, 2011). Aquatic species impacted by these oil spills exhibited cardiotoxicity that was correlated with the concentration of 3‐ringed PAH subfamilies such as phenanthrenes and fluorenes (Carls et al., 1999; Heintz et al., 1999). Subsequent work across a range of fish and invertebrate species has revealed that tricyclic PAHs exert their cardiotoxic effects by inhibiting ion currents in cardiomyocytes (e.g., fish (Abramochkin et al., 2021; Abramochkin et al., 2023; Ainerua et al., 2020; Brette et al., 2017; Kompella et al., 2021; Vehniäinen et al., 2019) and decapods (Ainerua et al., 2021; Dubiel et al., 2024)). These studies show the primary target is the major repolarizing potassium current, I_Kr_, whose suppression is proarrhythmic. Additionally, tricyclic PAHs can inhibit the sodium current (I_Na_), the main depolarizing current in cardiomyocytes (Abramochkin et al., 2021, 2023; Vehniäinen et al., 2019) as well as the L‐type calcium current (I_Ca_) (Ainerua et al., 2020; Kompella et al., 2021; Vehniäinen et al., 2019) and intracellular calcium cycling (Brette et al., 2017), which are crucial for cardiomyocyte contraction. Similar effects on human cardiomyocytes could have serious health consequences, especially in vulnerable populations such as people living with heart disease or in areas with highly polluted air.

Phenanthrene is the dominant tricyclic PAH in both the gaseous phase of air pollution and adsorbed to the surface of particulate matter (PM) (Abdel‐Shafy & Mansour, 2016). Phenanthrene is not routinely measured in ambient air monitoring, but a 1994 report found phenanthrene levels of 35.7 ng/m^3^ and 76–82 ng/m^3^ in Manchester and London, respectively (Halsall et al., 1994). A recent report found phenanthrene levels of up to 30.2 μg/m^3^ in ambient air associated with waste burning/recycling sites in Ghana (Kwarteng et al., 2022). Phenanthrene enters the body through inhalation and ingestion and can accumulate in body tissues (England et al., 2024). A recent Greek study found 2‐fold higher levels of phenanthrene compared with all other PAHs in human serum and 4‐fold higher levels of phenanthrene in heart failure patients (~1.3 μM) than in control subjects (0.14–0.58 μM) suggesting a link between serum concentration and disease (Chrysochou et al., 2021). Phenanthrene is also highly lipophilic (logP [n‐Octanol/water] coefficient of ~4.4) and can accumulate in fat at higher levels than in serum, as indicated from animal studies (Carls et al., 1999).

Phenanthrene interacts directly with wild‐type (WT) and mutant human *Ether‐à‐go‐go‐Related Gene* (hERG) channels at a noncanonical drug binding site, showing isoform‐dependent inhibition (IC_50_ = 17.6 ± 1.7 μM for I_hERG1a_, while IC_50_ = 1.8 ± 0.3 μM for I_hERG1a/1b_) (Al‐Moubarak et al., 2021). As hERG channels are essential for normal ventricular repolarization in humans, their inhibition following acute exposure may increase the risk of pollution‐associated arrhythmias. We recently investigated this possibility in mice and found that acute exposure to phenanthrene inhibited depolarizing (I_Ca_ and I_Na_) and repolarizing currents (I_to_ and I_Kur_) in ventricular myocytes, which manifest as PR prolongation in the mouse electrocardiogram, slower ventricular conduction velocity, and an overall increase in arrhythmia susceptibility recorded ex vivo (Yaar et al., 2023).

Here, we use healthy adult female sheep (*Ovis aries*) to probe the cardiotoxicity of phenanthrene in a large‐animal model. There are strong parallels between the human and sheep cardiovascular systems, including comparable gross heart structure (Perry et al., 2015), heart rate (Vehniäinen et al., 2016), and cardiac output (Stowe et al., 1985). Importantly, excitation‐contraction coupling in sheep and human cardiomyocytes is similar (Milani‐Nejad and Janssen, 2014) allowing comparison between systems. Based on observations in fish and mouse myocytes, and on the data from the hERG expression systems, we hypothesized that acute phenanthrene exposure would inhibit repolarizing potassium currents, prolong APD, and disrupt calcium handling through both transsarcolemmal and intracellular processes in sheep ventricular myocytes.

## 2 MATERIALS AND METHODS

### 2.1 Animal model

Reporting of animal experiments was in accordance with the ARRIVE guidelines 2.0. Adult female Welsh mountain sheep aged 18 months +/− 6 months were group‐housed in a facility maintained on a 12‐h light/12‐h dark cycle, and afforded ample bedding and a diet of hay. Only female sheep were used in this study as females are more amenable to living in the animal care unit than are males. All were free of observable disease and had acclimatized to the housing facility for a minimum of 1 week prior to starting the study.

### 2.2 Isolation of sheep ventricular cardiomyocytes

Sheep left ventricular myocytes were isolated as previously described (Briston et al., 2011; Caldwell et al., 2014). Briefly, sheep were euthanized by pentobarbitone (200 mg.kg^−1^; Pentoject, Animalcare Ltd) intravenous injection. Hearts were excised and rinsed in calcium‐free solution [composition (in mM): 134 NaCl, 4 KCl, 1.2 MgSo_4_, 1.2 NaH_2_PO_4_, 10 2,3‐Butanedione monoxime (BDM), 10 4‐(2‐hydroxyethyl)‐1‐piperazineethanesulfonic acid (HEPES), 11 Glucose and 0.5 mg/mL bovine serum albumin (BSA)] and dissected along the coronary sulcus, separating the atria and ventricle. The left anterior descending coronary artery was cannulated and perfused with calcium‐free isolation solution at 37°C for 10 mins at ~20 mL.min^−1^ (depending on the size of the heart). Next, the heart was perfused with the calcium‐free solution containing the digestive enzymes collagenase type II (0.096 mg.mL^−1^, cat no: LS004177, Worthington Biochemical Corporation) and protease type XIV (0.02 mg.mL^−1^, cat no: P5147, Sigma‐Aldrich) for 10 min. Lastly, a low‐calcium taurine solution was perfused through the heart for an additional 20 min [composition (in mM): 113 NaCl, 4 KCl, 1.2 MgSO_4_, 1.2 NaH_2_PO_4_, 10 2,3‐Butanedione monoxime (BDM), 10 HEPES, 11 Glucose, 50 taurine, 0.1 CaCl_2_, and 0.5 mg/mL bovine serum albumin (BSA)]. The digested left side of the heart was then dissected free from the rest of the myocardium and separated from the epicardium and endocardium. Individual cardiomyocytes were released by gentle mincing, trituration, and filtering through a 200 μm nylon mesh. After settling, myocyte pellets were resuspended in a 50:50 mix of Normal Tyrode's (NT) and taurine solution containing (in mM) 140 NaCl, 4 KCl, 1 MgCl_2_, 10 HEPES, 10 Glucose, and 1.8 CaCl_2_. 2 probenecid (cat no: P36400, Invitrogen) (2 mM) was included for fluorescence experiments. All solutions were pH adjusted to 7.34 with NaOH. Myocytes were stored at room temperature for up to 8 h.

All myocyte recordings were performed at ~37°C maintained via a combination of a Quick Exchange Heated Platform and in‐line heater system (Warner Instruments, LLC, CT, USA) delivering NT to myocytes from darkened glass syringes (Sanitex, Switzerland) using a gravity superfusion pinch‐valve system controlled by a VC‐6 six‐channel valve controller (Warner Instruments, LLC, CT, USA).

### 2.3 Phenanthrene

Phenanthrene solutions were made on the day of the experiments using phenanthrene (certified reference material grade, cat no: 73338, Sigma‐Aldrich) dissolved in DMSO to a 50 mM stock. From this stock, working solutions of 3, 10, and 25 μM were prepared in NT. All solutions were maintained in darkened reservoirs/tubes and light was dimmed during experimentation. Saline solutions were not gassed and were gravity delivered to the recording chamber from darkened glass tubes through polyetheretherketone HPLC tubing, which is narrow, chemically resistant, and inert. These precautions have been shown to reduce the loss of phenanthrene due to hydrophobicity during exposures (see; Yaar et al., 2023).

### 2.4 Calcium transient and membrane potential imaging

Isolated cardiomyocytes were either loaded with the calcium‐sensitive probe, Fluo‐4 am (cat no: F14201, Invitrogen), or the voltage‐sensitive probe, FluoVolt (cat no: F10488, Invitrogen) at room temperature. For Fluo‐4 am loading, myocytes were incubated with 0.5 μM Fluo‐4 am and 0.02% Pluronic F‐127 (cat no: P2443, Sigma‐Aldrich) for 4 min, followed by a 30‐min de‐esterification period. To load myocytes with FluoVolt, 1 mL of isolated cell suspension was incubated with 0.5 μL FluoVolt dye and 5 μL 100X PowerLoad Concentrate (cat no: P10020, ThermoFisher) for 20 min, followed by a 30‐min de‐esterification period. An aliquot of myocytes was placed into a glass well (cat no: 81158, Ibidi) on the stage of an inverted epifluorescence microscope (Nikon eclipse TE2000‐U, Nikon) and allowed to settle before being superfused with 37°C NT and stimulated to contract via field stimulation at 0.5 Hz with platinum wire electrodes with a 10 ms duration, at 70–100 V. A stimulation frequency of 0.5 Hz was chosen for this study as myocytes produced consistent amplitude transients over the duration needed for control and exposure measures to be conducted (~7 min). At stimulation frequencies closer to in vivo rates of 1–2 Hz, myocytes fatigued more rapidly. The probes were excited at 488 nm, and emission signals were collected between 510 and 560 nm. Signals were acquired using a PMT coupled to an OptoScan monochromator (Cairn Instruments, UK) connected to a Digidata 1440A with Clampex 11 acquisition software (Molecular Devices, USA).

To eliminate concerns of DMSO specific effects, control calcium transients and APs were recorded in NT solution containing DMSO at the concentration that matched the DMSO concentration used in the exposure solution for that cell (maximum DMSO was 1:2000). Thus, each recording was made in a given cell under these control conditions, and then again from the same cell after an 8‐min superfusion with either the same saline (time‐matched control, TMC) or with phenanthrene at either 3, 10, or 25 μM. During the 8 min superfusion, the cell was not illuminated or stimulated to contract. After exposure, cell stimulation and illumination recommenced for a series of 10–15 transients or APs to reach steady state. Measurements were taken from the last 3 to 4 waveforms after attainment of steady state.

To assess the impact of phenanthrene exposure on intracellular calcium cycling through the sarcoplasmic reticulum (SR), Fluo‐4 am loaded myocytes were preincubated for 30 min prior to the start of the experiment with 2 μm thapsigargin (cat no: 1138, Tocris) to inhibit sarco(endo)plasmic reticulum calcium ATPase (SERCA) activity and 10 μM ryanodine (cat no 1329, Tocris) to inhibit SR calcium release channels (ryanodine receptors) and then calcium transients were recorded before and after phenanthrene exposure as described above. In some experiments, SR inhibition was achieved through direct superfusion on the recording stage rather than via preincubation, as specified in the figure legends.

### 2.5 Whole‐cell patch‐clamp

Patch‐clamp electrophysiological data were recorded as previously described (Kompella et al., 2021). Briefly, myocytes were allowed to settle before each trial for ~2 min on ibidi glass bottom 35 mm dishes (cat no. 81218‐200, Ibidi) sat within the Quick Exchange Heated Platform (Warner Instruments) which contains heating elements and holds superfusion and suction tubes. Following this, myocytes were perfused with NT, and cells were patched, and whole cell configuration was attained. Following recordings under control conditions, the perfusion was switched to one containing phenanthrene. The maximum final DMSO concentration used in drug solutions was 0.05%, corresponding to 25 μM phenanthrene solution. Preliminary studies showed no impact of the DMSO vehicle on the ion channel measurements; thus, we did not express data relative to DMSO controls for electrophysiological recordings. All recordings were performed at 35 ± 2°C using the heated platform and superfusion system described above. Data were recorded via a Digidata 1322A A/D converter (Axon Instruments). Signals were filtered at 2 kHz using an 8‐pole Bessel low pass filter before digitization at 10 kHz and storage. Patch pipette resistance was typically 3.5–4 MΩ when filled with intracellular solution. Series resistance ranged between 5 and10 MΩ and was compensated up to 60% for I_CaL_ voltage clamp experiments (voltage error ≤2 mV).

The rapid delayed rectifier potassium channel current (I_Kr_) was recorded using NT solution containing nifepidine (cat no: 481981, Sigma) (20 μM) to inhibit L‐type calcium channels. The I_Kr_ pipette solution contained (in mM): 10 NaCl, 140 KCl, 5 MgATP, 5 EGTA, 1 MgCl_2_, and 10 HEPES, pH adjusted to 7.2 with KOH. I_Kr_ was activated by a test‐pulse to 0 mV (2.5 s) to fully activate potassium channels (as determined in preliminary experiments) and measured as the tail current at −60 mV (4.5 s) at a frequency of 0.1 Hz. A pre‐pulse to −40 mV from holding potential of −60 (1 s) was used to inactivate any sodium and T‐type calcium current. The tail currents were confirmed as I_Kr_ following the superfusion of 2 μM E‐4031 (selective I_Kr_ inhibitor) in preliminary experiments (representative trace shown in Figure 2a and bar graph in Figure 2b).

**TABLE 1:** Absolute values for FluoVolt measurements (ms).

	Control	Exposure dose	Phenanthrene	*n* (*N*)
APD_30_	192.9 ± 48.2	DMSO TMC	182.6 ± 55.7	7 (5)
	182.3 ± 28.7	3 μM Phe	177.6 ± 33.8	9 (6)
	195.0 ± 57.2	10 μM Phe	188.1 ± 60.2	9 (6)
	181.3 ± 58.7	25 μM Phe	113.8 ± 73.4	11 (6)
APD_50_	256.4 ± 58.3	TMC	267.2 ± 69.4	6 (5)
	245.1 ± 42.1	3 μM Phe	240.4 ± 48.7	9 (6)
	273.6 ± 76.2	10 μM Phe	260.5 ± 79.4	9 (6)
	244.2 ± 71.0	25 μM Phe	142.5 ± 91.1	11 (6)
APD_90_	357.5 ± 75.6	TMC	365.9 ± 81.7	6 (5)
	350.7 ± 61.0	3 μM Phe	356.1 ± 82.0	9 (6)
	394.7 ± 92.6	10 μM Phe	388.1 ± 96.3	9 (6)
	349.7 ± 96.7	25 μM Phe	215.3 ± 119.3	11 (6)

*Note*: APs were recorded under control conditions and then following exposure to either a time matched control (DMSO TMC, time‐matched control) or phenanthrene at either 3 μM, 10 μM, or 25 μM. *p* values are provided in Figure 1.

For L‐type calcium current (I_CaL_) measurement, KCl in NT solution was replaced with CsCl to inhibit potassium channels, and the currents were elicited using a voltage protocol that consisted of a test pulse from −40 mV to 0 mV for 300 ms and a pre‐pulse to −40 mV from a holding potential of −80 mV (2 s) to inactivate sodium and T‐type calcium current. The stimulus frequency was 0.1 Hz. The I_CaL_ pipette solution contained (in mM) 130 CsCl, 15 TEA‐Cl, 5 MgATP, 1 MgCl_2_, 5 Na_2_‐phosphocreatine, 5 EGTA, 10 HEPES, and 0.03 Na_2_GTP, pH adjusted to 7.2 with CsOH. CsCl and TEA‐Cl were included to inhibit K+ currents.

### 2.6 Data analysis

Data were analyzed offline using Clampfit 11.0.3 (Axon instruments). From the calcium transients, several parameters were measured from the fluorescent signal: baseline, peak amplitude (as measured by the difference between baseline and peak amplitude normalized to its respective baseline, ΔF/F0), decay rate (as measured as tau), and time to peak.

Tau (τ) was fit using the experimental standard equation:

$$f\left(t\right)=\sum_{i=1}^{n}A_{i}e^{-t/\tau_{i}}+C$$

For the membrane potential study, to measure action potential duration (APD), an average trace was created using Clampfit 11.0.3 following the attainment of steady‐state, and voltage values were normalized, such that the baseline was 0 V and the peak of the AP was 1 V. The time taken for 30% (0.8 V), 50% (0.5 V), and 90% (0.1 V) repolarization was determined, termed APD_30_, APD_50_, and APD_90_, respectively. Paired data have been reported as a percentage change from the control recording and displayed as mean ± SD in figures and text unless stated otherwise. For most analyses, a nested linear mixed model was used to allow for the inclusion of both fixed (phenanthrene exposure) and random (individual animal) effects, as described in the figure legends.

Electrophysiological data was also analyzed with Clampfit 11.0.3. Patch‐clamp data are reported as mean ± SD, and currents are expressed as current density by normalizing to cell size (pApF^−1^). I_Kr_ tail currents were measured as the difference between the peak and the end of the repolarizing pulse current. I_CaL_ amplitude was measured as the difference between the peak and the end of the depolarizing pulse current. The inactivation rate of I_Ca_ was quantified by fitting inactivation with the following bi‐exponential equation:

$$\text{34𝐼}={\text{34𝐴}}_{\text{34𝐴𝑓}}\;{\text{34𝑒}}^{\left(-\text{𝑡}/\text{34𝜏34𝜏𝑓}\right)}+{\text{34𝐴}}_{\text{𝑠}}\;{\text{34𝑒}}^{\left(-\text{𝑡}/\text{34𝜏𝑠}\right)}+\text{34𝐶}$$

where, *I* represents the current amplitude at time *t*; *A*_*f*_ and *A*_*s*_, respectively, represent the amplitude of fast and slow inactivating current components, fitted with time constants *τf* and *τs*; *C* represents any residual unfitted current. Due to a non‐normal distribution, paired data were analyzed with the Wilcoxon signed rank test (percentage data) or a Mann–Whitney test with *p* values as indicated within the text and figure legend using GraphPad Prism 8 software. Sample sizes for animals are given as *N* and for myocytes as *n* in text and figure legends. Statistical analysis used and *p* values are included in the figure legends and in the text.

## 3 RESULTS

### 3.1 Acute phenanthrene exposure shortens the action potential duration at high doses

**FIGURE 1:**
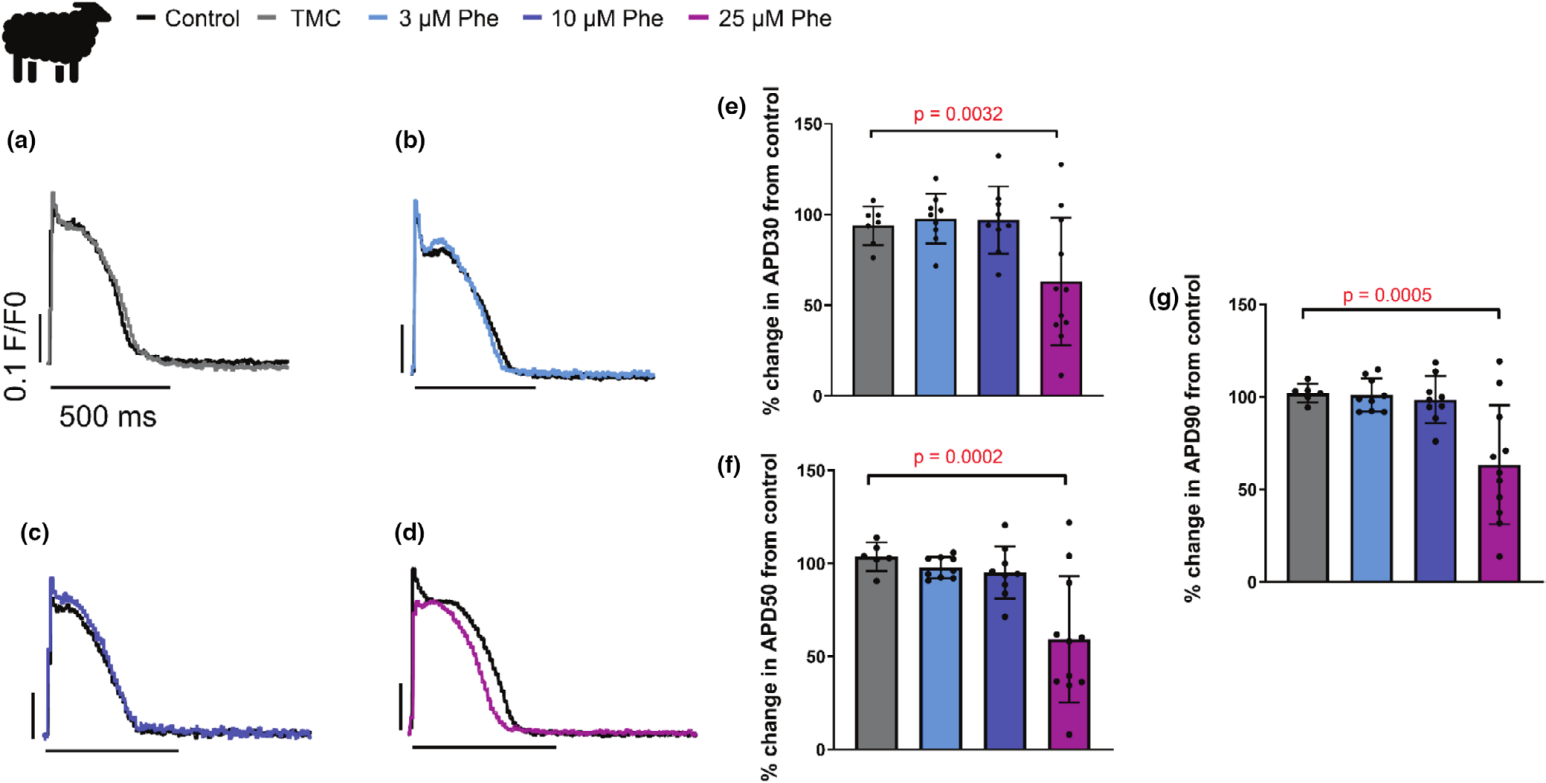
Phenanthrene reduces action potential duration in sheep ventricular cardiomyocytes. (a–d) Representative optical action potentials (APs) recorded from isolated ventricular cardiomyocytes loaded with FluoVolt and field stimulated at 0.5 Hz. All data are paired. APs were recorded under control conditions (control, black) and compared to either a time matched control (DMSO TMC, time‐matched control, gray) or phenanthrene at either 3 μM (light blue), 10 μM (blue), or 25 μM (purple). The time scale is 500 ms for all traces. We show the extended time duration of the optical AP to show the recordings were made at steady‐state. (e–g) Bar graphs show means ± SD of % change in action potential duration (APD) from control at (e) 30%, (f) 50%, and (g) 90% repolarization. *N* = 12 sheep; *n* = 6–11 myocytes per treatment, *p* values from nested linear mixed model. TMC, time‐matched control. Absolute values are shown in Table 1.

Phenanthrene had no effect on APD at 3 or 10 μM (Figure 1). At 25 μM exposure, the APD was significantly shortened at 30% (APD_30_, *p* = 0.0032; *N* = 12; *n* = 6–11), 50% (APD_50_, *p* = 0.0002; *N* = 12; *n* = 6–11) and 90% (APD_90_, *p* = 0.0005; *N* = 12; *n* = 6–11) repolarization when compared to myocytes only exposed to DMSO time‐matched control (TMC) (Figure 1). APD_30_ was reduced to 63.0 ± 35.1% (*N* = 6; *n* = 11), APD_50_ to 59.2 ± 34.0% (*N* = 6; *n* = 11) and APD_90_ to 63.5 ± 32.2% (*N* = 6; *n* = 11) of its time‐matched control after acute exposure to 25 μm phenanthrene (Figure 1). Although optical APs can be poor at resolving absolute voltages, we compared the baseline fluorescence (reflecting resting membrane potential and the upstroke velocity of the AP before normalization) and found no significant effect of phenanthrene on either parameter (Baseline florescence; DMSO TMC, 0.067 ± 1.48 AU and 25 μM phenanthrene, 0.007 ± 0.011 AU, *n* = 14, Upstroke velocity; DMSO TMC, 1.29 ± 0.71 AU versus 25 μM phenanthrene, 1.20 ± 0.74 AU, *p* = 0.128, paired *t*‐tests).

### 3.2 Acute phenanthrene exposure inhibits I_Kr_ but not I_Ca_

**FIGURE 2:**
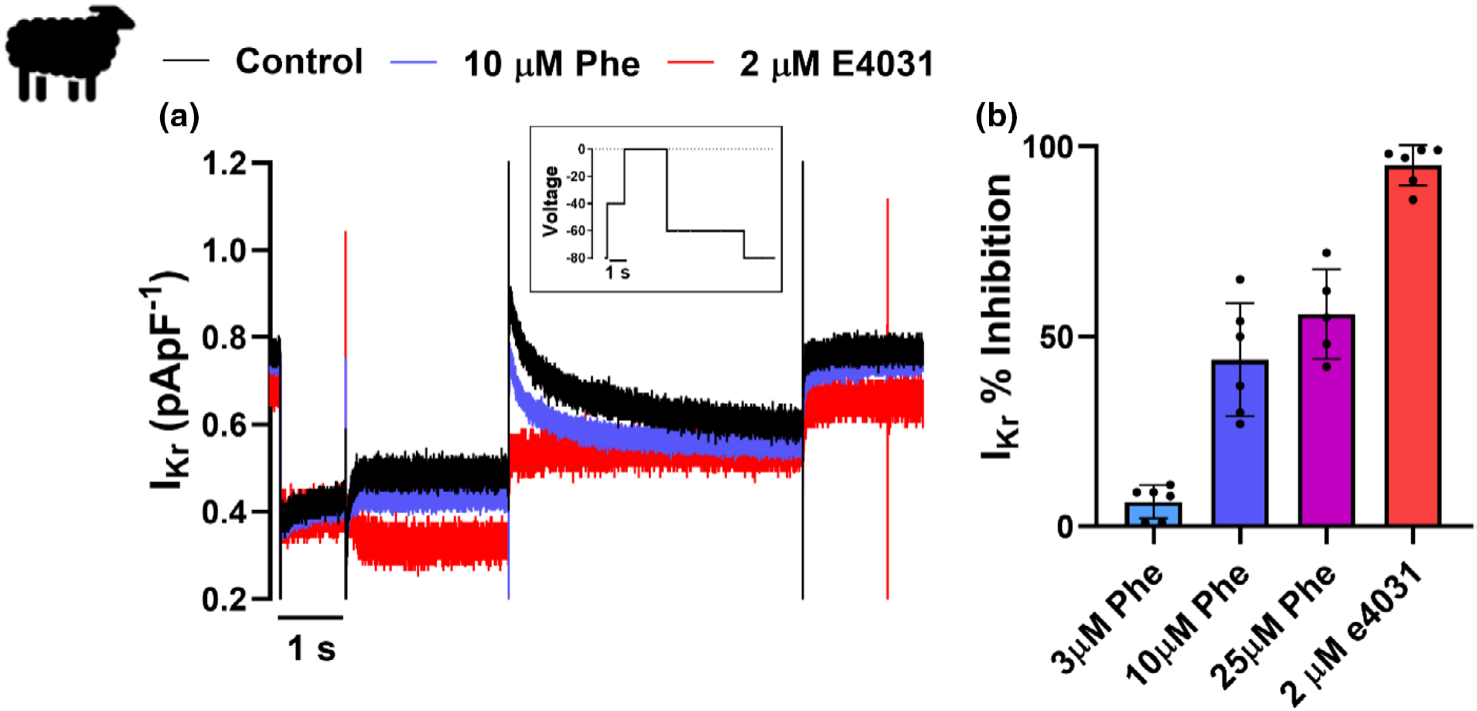
Sheep I_Kr_ tail‐current is significantly inhibited by phenanthrene exposure. (a) Whole‐cell patch‐clamp recording of rapid delayed rectifier potassium channel current (I_Kr_) density before (black trace), after acute exposure to 10 μM phenanthrene (Phe) (blue trace), and following block by 2 μM E4031, a specific inhibitor of I_Kr_ (red trace). I_Kr_ tail current was elicited at −60 mV (4.5 s) following a test pulse to 0 mV (2.5 s). A pre‐pulse to −40 mV for 1 s was used to inactivate any sodium and T‐type calcium current (see inset). (b) The bar graph shows the percentage inhibition of I_Kr_ tail current (mean data ± SD) compared to the control in the presence of phenanthrene (3 μM Phe—6.5% ± 4.4; 10 μM Phe—43.8% ± 14.9; 25 μM Phe—55.8% ± 11.8) or e4031 (selective I_Kr_ inhibitor) (95% ± 5.3). *N* = 3–4 sheep; *n* = 5–6 myocytes. Inhibition was significant at all doses, as provided for the absolute current data in Table 2.

**FIGURE 3:**
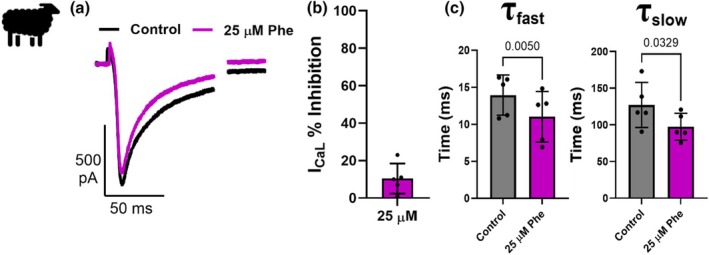
Phenanthrene exposure alters calcium current (I_Ca_) inactivation kinetics but does not affect the peak amplitude. (a) Whole‐cell patch‐clamp recording of L‐type calcium channel current (I_Ca_) from sheep left ventricular cardiomyocytes, measured during a test pulse from −40 mV to 0 mV for 300 ms (holding potential of −80 mV; a pre‐pulse to −40 mV from holding potential was used to inactivate any sodium and T‐type calcium current) in the absence (black, control) and presence (purple, 25 μM Phe) of 25 μM phenanthrene (Phe). (b) Mean data ± SD of the inhibition of I_Ca_ expressed as percentage change of control shows no significant difference (10.4% ± 8.1; *N* = 3; *n* = 5) (*p* = 0.052; two‐tailed paired *t*‐test). (c) Bar graphs of mean ± SD showing a significant decrease in the time constant of fast (*τ_f_*) inactivation (Control—13.97 ± 2.7 ms; 25 μM Phe—11.03 ± 3.4 ms; *N* = 3; *n* = 5; *p* = 0.005, two‐tailed paired *t*‐test) and slow (τ_s_) inactivation (Control—127.1 ± 30.7 ms; 25 μM Phe—97.35 ± 18.3 ms; *N* = 3; *n* = 5; *p* = 0.033, two‐tailed paired *t*‐test) of calcium current with exposure to 25 μM phenanthrene (Phe).

Significant inhibition of I_Kr_ was observed at 3 μM (*p* = 0.0301; *N* = 4; *n* = 6), 10 μM (*p* = 0.0313; *N* = 4; *n* = 6) and 25 μM phenanthrene (*p* = 0.0002; *N* = 3; *n* = 5) with a maximum inhibition of 55.8 ± 5.3% at 25 μM (Figure 2, Table 2). Inhibition of I_Kr_ would be expected to progressively prolong APD with ascending doses. However, we observed shortening of APD at 30, 50, and 90% repolarization in this study, and only at the highest dose of phenanthrene (25 μM). We therefore next investigated phenanthrene's impact on the key inward current during the AP plateau, I_Ca_, and found only a minor (10.4 ± 3.6%, *p* = 0.0519; *N* = 3; *n* = 5) inhibition, which was not significant, even at the highest concentration of phenanthrene (Figure 3). Interestingly, 25 μM phenanthrene exposure significantly decreased the time constant of fast (*τ*_*f*_) and slow (*τ*_*s*_) inactivation (Figure 3b). However, no significant change was seen for the fraction of fast and slow amplitude of the inactivation kinetics (*A*_*f*_: control—0.915 ± 0.03, 25 μM phenanthrene—0.92 ± 0.04; *A*_*s*_: control—0.085 ± 0.02, 25 μM phenanthrene—0.08 ± 0.04, not shown).

**TABLE 2:** I_Kr_ tail‐current density (pApF^−1^).

Control	Exposure dose	Phenanthrene	*p* Value	*n* (*N*)
0.48 ± 0.1	3 μM Phe	0.45 ± 0.09	0.03	6 (3)
0.40 ± 0.05	10 μM Phe	0.23 ± 0.05	0.0312	6 (4)
0.37 ± 0.02	25 μM Phe	0.17 ± 0.03	0.0002	5 (4)
0.39 ± 0.02	2 μM e4031	0.02 ± 0.01	0.0312	6 (4)

*Note*: Currents were recorded under control conditions and then following the application of only a single dose of phenanthrene or the specific I_Kr_ inhibitor e4031. Significance *p* values were obtained using a paired *t*‐test for 3 and 25 μM Phe and a Wilcoxen test for 10 μM Phe and 2 μM e4031, depending on the normality of the data as assessed by the Shapiro–Wilk test.

### 3.3 Phenanthrene disrupts calcium transients

**FIGURE 4:**
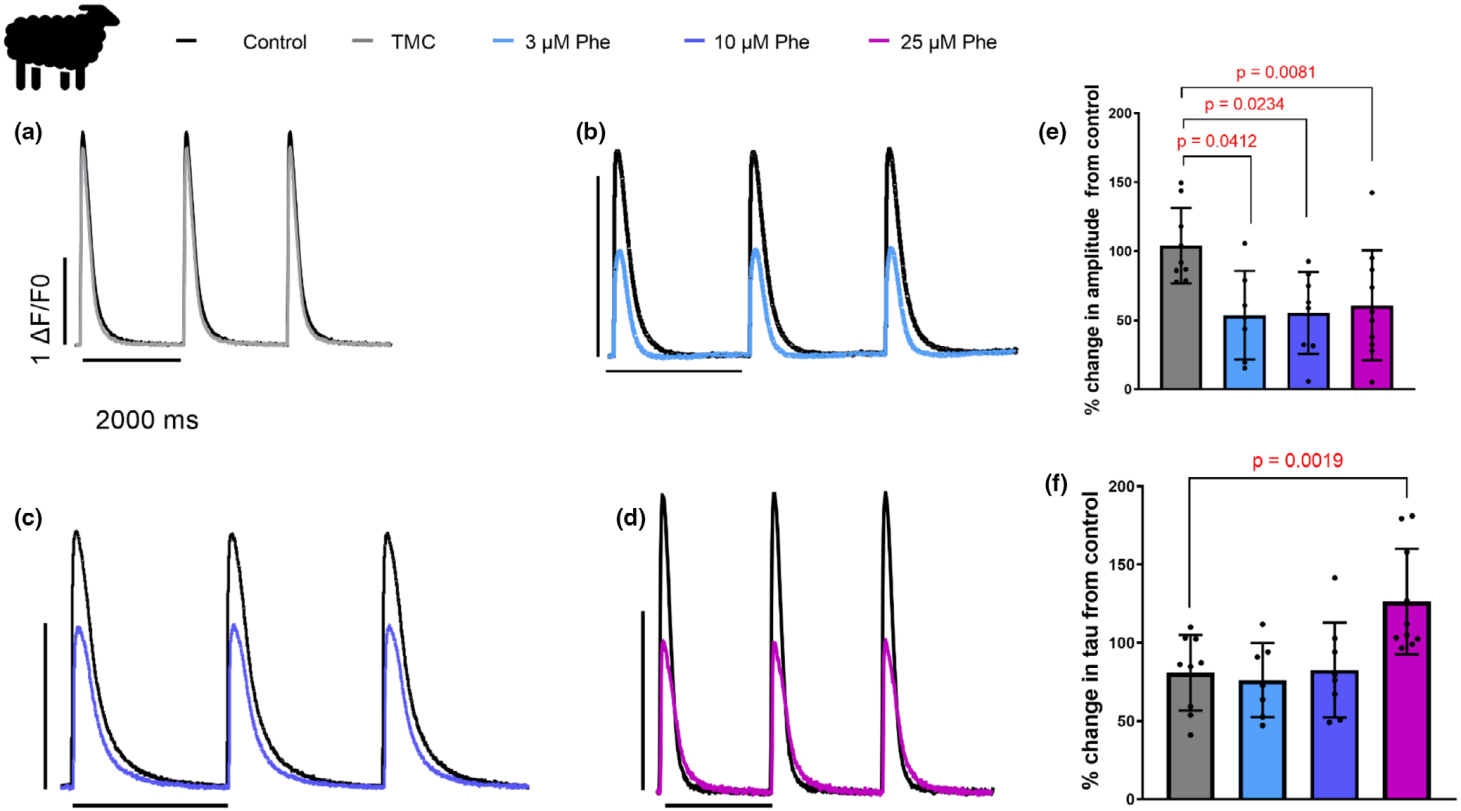
Phenanthrene disrupts intracellular calcium movement in sheep ventricular cardiomyocytes. (a–d) Calcium transients recorded from Fluo‐4am loaded ventricular cardiomyocytes before (control, black) and compared to either a time matched control (DMSO TMC, time‐matched control, gray) or phenanthrene at either 3 μM (light blue), 10 μM (blue), or 25 μM (purple). (e) Amplitude was calculated as change in fluorescence over baseline fluorescence (ΔF/F0) and expressed as percentage change from control transients. Data show means ± SD. (f) Rate of decay was measured using the time constant tau (time taken to decay to 37% of its peak) and expressed as percentage change from control transients. *N* = 9; *n* = 7–10, *p* values are provided assessed with nested linear mixed model.

Using the calcium‐sensitive probe Fluo‐4 am, we found significant reductions in the amplitude of the intracellular calcium at all concentrations of phenanthrene (3 μM *p* = 0.0412, 10 μM *p* = 0.0234, and 25 μM *p* = 0.0081, *N* = 9; *n* = 7–10) compared to the DMSO TMC (Figure 4a–e). Amplitudes were reduced to 53.7 ± 32.0% (*N* = 4; *n* = 7) of its control by 3 μm phenanthrene, 55.3 ± 29.7% (*N* = 5; *n* = 8) by 10 μm phenanthrene, and 60.7 ± 39.9% (*N* = 4; *n* = 10) by 25 μm phenanthrene. There were no significant differences in diastolic calcium between the groups (TMC baseline florescence AU 1.314 ± 1.345 vs. 25 μm phenanthrene baseline florescence AU 0.8747 ± 0.5164, *N* = 4, *n* = 10, *p* = 0.099, paired *t*‐test), indicating that these changes were due to a reduction in systolic calcium levels. The rate of decay of the transient, measured as the time constant tau, was prolonged by 26.4 ± 33.6% after acute exposure to 25 μM phenanthrene (Figure 4f), significantly greater than that of the DMSO TMC exposed cells (*p* = 0.0019; *N* = 5; *n* = 9–10) Tau was not impacted at lower phenanthrene exposure doses. No changes were observed in the time to peak of the calcium transient following phenanthrene exposure at any dose (not shown).

### 3.4 Sarcoplasmic reticulum calcium cycling is impacted by phenanthrene

**FIGURE 5:**
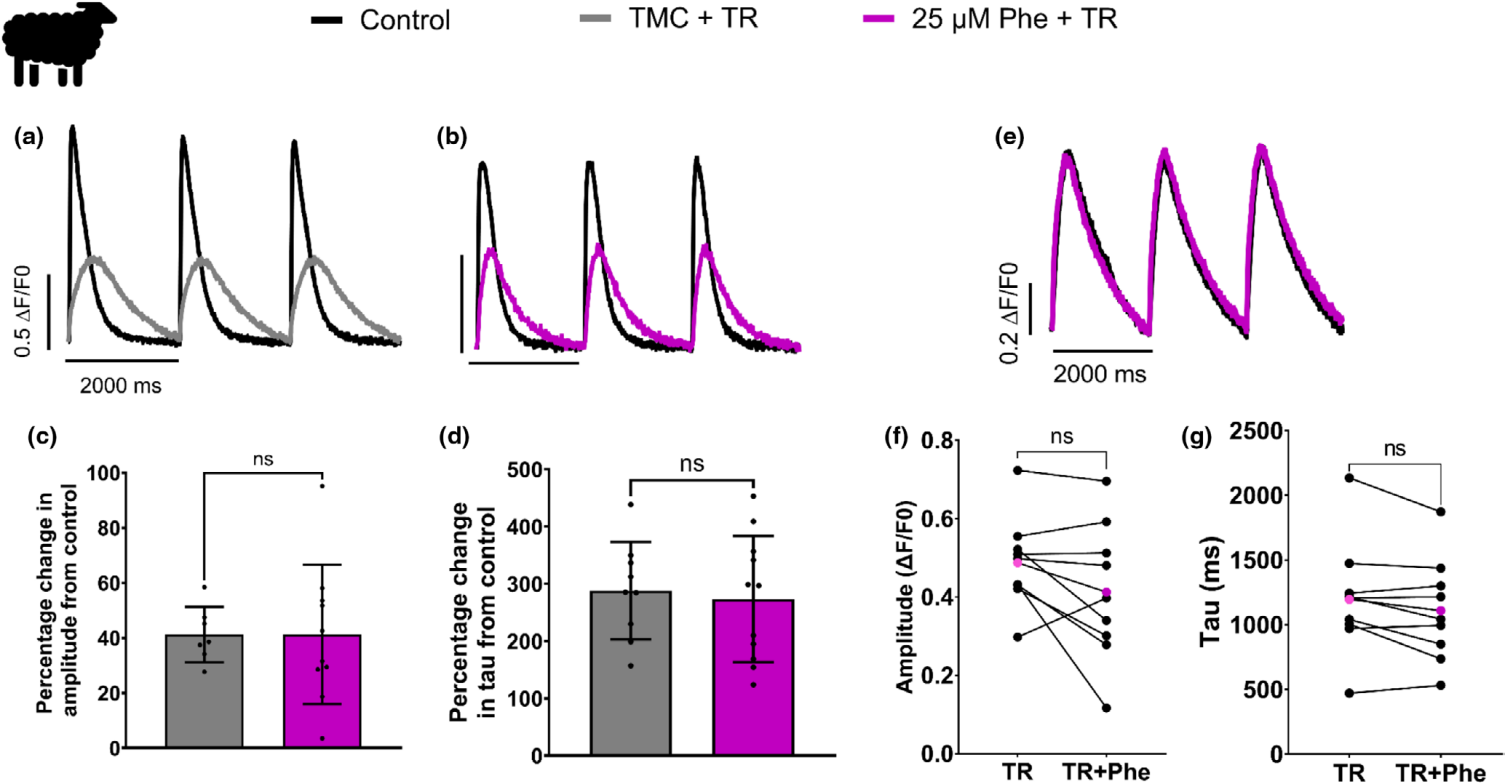
Inhibiting the sarcoplasmic reticulum (SR) abolishes phenanthrene's impact on the intracellular calcium handling in sheep ventricular myocytes. (a and b) Representative traces of calcium transients recorded from sheep ventricular myocytes before (black, control) and after the addition of either DMSO with 2 or 5 μM thapsigargin and 1 or 10 μM ryanodine (TR) (gray, DMSO TMC + TR) or 25 μM phenanthrene with TR (purple, 25 μM Phe + TR). Mean data ± SD expressed as percentage change from control for amplitude (ΔF/F0) (c) and tau (d) (*N* = 6; *n* = 9–11; ns = no significance, Mann–Whitney test). (e) Representative calcium transients recorded after a 30‐min preincubation with 2 μM thapsigargin and 10 μM ryanodine (black line), and then after acute exposure to phenanthrene (purple, 25 μM Phe + TR). Graphs showing amplitude (ΔF/F0) (f) and tau (g) from individual preincubated myocytes before (left side) and after acute 25 μM phenanthrene exposure (plus TR) (right side). Purple dots indicate the mean of the data. *N* = 2; *n* = 9; ns = no significance, paired *t*‐test. TMC, time‐matched controls.

The exposure‐induced change in calcium transient amplitude and rate of decay, in the absence of a change in transsarcolemmal flux through I_Ca_, implicates altered sarcoplasmic reticulum (SR) calcium cycling. However, changes in AP shape may also be involved. To understand the role of SR calcium cycling in the response to phenanthrene, we next used two pharmacological SR inhibitors: ryanodine, an inhibitor of the SR calcium release channel/ryanodine receptor RYR2, and thapsigargin, an inhibitor of the SERCA, to investigate the impact of phenanthrene on SR cycling. Calcium transients were first recorded under control conditions and then following 8 min superfusion with NT saline containing thapsigargin and ryanodine. SR inhibition caused a reduction in the calcium transient amplitude and a slowing in the rate of decay compared with control (41.3 ± 10.1% reduction in amplitude, 288.1 ± 85.1% prolongation in tau *N* = 6; *n* = 9; see gray lines/bars DMSO TMC + TR in Figure 5a,c,d). Next, control calcium transients were compared with myocytes perfused with thapsigargin, ryanodine, and 25 μM phenanthrene together. There was no additional decrease in the amplitude of the calcium transient (*p* = 0.8238, *N* = 6; *n* = 9–11) or in the time course of decay (*p* = 0.8125, *N* = 6; *n* = 9–11) compared with cells treated with just thapsigargin and ryanodine (41.3 ± 25.4% reduction in amplitude, 273.4 ± 110.0% prolongation in tau; *N* = 6; *n* = 11; see purple lines/bars in Figure 5b–d).

To ensure that we were recording calcium transients after maximal SR inhibition, myocytes were also preincubated with 2 μM thapsigargin and 10 μM ryanodine for 30 min, and then calcium transients were recorded during superfusion with SR inhibitors (TR). The same cell was then acutely exposed to 25 μM phenanthrene (with SR inhibitors, TR), and transients were recorded after 8 min. No change in calcium amplitude or time constant of decay was observed following acute phenanthrene exposure (amplitude *p* = 0.118; tau *p* = 0.0925; *N* = 2; *n* = 9) (Figure 5e–g). These data suggest that much of phenanthrene's effect on calcium handling in the sheep ventricular myocyte is due to disruption of SR calcium cycling.

## 4 DISCUSSION

The air pollutant phenanthrene has been shown to induce 11 out of the 12 recognized key characteristics of cardiovascular toxicity, including impaired contractility, impaired excitability, endothelial dysfunction, altered vascular homeostasis, and the induction of oxidative stress and inflammation (England et al., 2024). This characterization warrants the routine monitoring and reporting of phenanthrene levels as part of air quality assessment due to its health risk for humans and other animals. However, presently, governmental agencies across the world do not monitor tricyclic PAHs specifically, and indeed, many do not even routinely report total PAH levels. This inaction may be due, at least in part, to a lack of cardiotoxicity data from humans or from a large animal model that reflects human cardiovascular physiology. Here we demonstrate that acute exposure to phenanthrene at high, but realistic doses (3–10 μM, (Carls et al., 1999; Chrysochou et al., 2021)), has deleterious impacts on excitation–contraction coupling processes in ventricular myocytes from the sheep, a large mammalian model with translational relevance to human cardiac physiology. We show APD shortening at the highest doses (25 μM) and I_Kr_ inhibition and disruption of the calcium transient through SR calcium cycling at lower doses (3–10 μM). The following discussion compares our findings in the sheep to what is known for other species to better appreciate the ubiquity of this cardiotoxicant. Studies with fish have provided much of the foundational work on the impacts of PAHs on vertebrate myocardial function due to the widespread pollution of the aquatic environment with PAHs from oil spills (Marris et al., 2020). Fish are a relevant model for understanding mammalian toxicity due to the similarities between fish and human AP characteristics (Ravens, 2018). We hope the findings of this large animal study provide the impetus necessary to raise awareness and ignite an investigation into cardiac health issues for those living in areas with poor air quality.

### 4.1 Myocyte electrical activity

The inhibitory effect of phenanthrene on I_Kr_ is species‐specific, but EC_50_ values are usually in the low micromolar range. The EC_50_ for I_Kr_ measured in isolated cardiomyocytes from navaga cod, zebrafish, brown trout, bluefin tuna, and rainbow trout are ~2.2 (Abramochkin et al., 2021), 3.3 (Kompella et al., 2021), 7 (Ainerua et al., 2020), 5 (Brette et al., 2017), and 10 μM (Vehniäinen et al., 2019), respectively; EC_50_ for shorthorn sculpin is higher at 144 nM (Filatova et al., 2022). This is the first study we know of to report inhibition of I_Kr_ in native mammalian myocytes. We could not calculate the EC_50_ here, but the ~56% inhibition at the highest dose (25 μM) suggests the sheep I_Kr_ current is less sensitive to phenanthrene than that reported for aquatic ectotherms. Recombinant I_hERG_ carried by hERG1a channels expressed in HEK293 was also less sensitive to phenanthrene (EC_50_ ~ 17 μM), but when co‐expressed as hERG1a/1b, which is thought to underlie native mammalian I_Kr_ (Jones et al., 2004; Sale et al., 2008), EC_50_ increased to ~1.8 μM (Al‐Moubarak et al., 2021). Mouse cardiomyocytes do not have I_Kr_ current, with the fast repolarization in small mammals being carried by I_kur_ and I_to_ (Brouillette et al., 2004). Our previous work has shown both I_kur_ and I_to_ are sensitive to inhibition by phenanthrene (a decreased peak current amplitude of ~53% for I_Kur_ and by 37% for I_to_). Although I_Kr_ is the main repolarizing current in sheep (Brouillette et al., 2004; Verkerk et al., 2000), I_to_ does contribute to the early repolarization notch, but we did not isolate it or investigate the impact of phenanthrene on it in this study. We also did not investigate the slow repolarizing current (I_Ks_) in this study. It is not known if I_Ks_ is phenanthrene‐sensitive in any species, but as it plays a significant role in repolarization reserve, future work should investigate its potential contribution to APD shortening following phenanthrene exposure and I_Kr_ inhibition in the sheep.

Strong inhibition of I_Kr_ is expected to prolong APD, which is what is observed in most fish (Abramochkin et al., 2021; Ainerua et al., 2020; Brette et al., 2017) and in mouse (Yaar et al., 2023) following acute phenanthrene exposure. However, in contrast to our hypothesis, here in sheep ventricular myocytes, we observed little impact of phenanthrene exposure on APD except at the highest dose (25 μM), where APD shortened by ~37%. This coincides with comparable work on zebrafish, where significant shortening of APD_50_ and APD_90_ was observed at 3 and 10 μM phenanthrene exposure (Kompella et al., 2021). The shortening of AP in zebrafish was in part attributed to lower inhibition of I_Kr_ repolarizing current under AP‐like voltage protocol compared to I_Kr_ tail current voltage protocol (Kompella et al., 2021). The AP‐like voltage protocol could not be carried out in our study due to the surprisingly low I_Kr_ current density of 0.4 ± 0.04 pApF^−1^ in sheep ventricular myocytes (Figure 2b). The primary driver behind APD shortening in zebrafish was the large inhibitory effect (>50% inhibition at maximal dose) of phenanthrene on I_Ca_ (Kompella et al., 2021). Sheep and zebrafish ventricular myocytes both have a distinct plateau phase (Vornanen & Hassinen, 2016) produced in large part by the depolarizing L‐type calcium current (I_Ca_), which counteracts the outward flow of potassium (Verkerk et al., 2000). However, contrary to the zebrafish, we found no significant impact of phenanthrene on I_Ca_ in the sheep ventricle. Low sensitivity of I_Ca_ to phenanthrene has been reported for the navaga cod (Abramochkin et al., 2021) and brown (Ainerua et al., 2020) and rainbow trout (Vehniäinen et al., 2019), whereas I_Ca_ in other fish species, including zebrafish (Kompella et al., 2021), and members of the Scombridae family (Brette et al., 2017) is more sensitive. Thus, the potency of phenanthrene on I_Ca_ also appears to be species‐specific and can impact exposure‐associated changes in APD. The mechanism through which phenanthrene inhibits the L‐type calcium channel is currently unknown for any species; mutagenesis and computational docking studies would be useful.

We did not investigate the impact of phenanthrene on sodium channel conductance (I_Na_) in the current study. However, AP upstroke velocity following phenanthrene exposure in our optical action potential measurements was not significantly altered, suggesting I_Na_ is not sensitive to phenanthrene in sheep. However, electrophysiology has superior temporal resolution compared with optical methods, and thus this finding should be interpreted cautiously until confirmed with either patch clamp or microelectrode studies. In fish cardiomyocytes, the response of I_Na_ to phenanthrene has been variable. In rainbow trout, phenanthrene increases I_Na_ density (by ~10%) and increases the upstroke velocity of the AP (Vehniäinen et al., 2019), whereas in navaga cod, it decreases I_Na_ (~17% inhibition) (Abramochkin et al., 2021) and has no impact on AP. We did not observe any visible shift in the baseline of the optical action potentials in this study, suggesting a lack of impact of phenanthrene on resting membrane potential. Although this too requires cautious interpretation, it should be noted that in all studies conducted to date, I_K1_ has been insensitive to phenanthrene (Abramochkin et al., 2021; Kompella et al., 2021; Vehniäinen et al., 2019). The sodium‐calcium exchanger (NCX) can also contribute to myocyte electrical activity, but we did not measure its response to phenanthrene exposure in our study. Indeed, to our knowledge, no published studies to date have investigated the impact of phenanthrene on I_NCX_.

APD shortening could arise due to a reduction in depolarizing currents during the plateau or an increase in repolarizing currents. Our data show little impact on depolarizing calcium current and a large reduction in repolarizing K‐current, which should result in APD prolongation. However, at the highest doses tested (25 uM), where I_Kr_ is inhibited by 56%, we see shortening at 30%, 50%, and 90% repolarizations. Shortening at APD_30_ is not consistent with inhibition of I_to_, which has been demonstrated to be sensitive to phenanthrene in mice but was not tested in this study. The shortening at APD_50_ and APD_90_ could suggest that I_Kr_ is not the main repolarizing current in sheep myocytes. However, although the presence of I_Ks_ has not been established for sheep ventricular myocytes, the duration of the field‐stimulated AP (<500 ms) is not expected to be long enough to activate a significant I_Ks_ current (Verkerk et al., 2001), although this should be tested explicitly. Ca^2+^‐activated Cl^−^ currents have been shown to be important in arrhythmogenesis in sheep ventricular myocytes but are generally not thought to be active under basal conditions (Hiraoka et al., 1998). Rather, they are implicated in rapid repolarization (EADs) following adrenergic stimulation and result in transient repolarizations during the plateau phase of action potentials (Verkerk et al., 2000, 2001). It is possible that phenanthrene impacts these currents, and future work should test this explicitly.

### 4.2 Myocyte calcium handling

Despite the marginal inhibition of transsarcolemmal calcium influx through I_Ca_ inhibition, we observed that phenanthrene concentrations as low as 3 μM significantly reduced calcium transient amplitude (Figure 4). We are not aware of previous studies in mammals that have examined the impact of phenanthrene on the properties of the intracellular calcium transient. In fish, only two studies have examined this; both report a reduction in calcium transient amplitude following phenanthrene exposure (tunas (Brette et al., 2017) and trout (Ainerua et al., 2020)).

The marginal effect of phenanthrene on I_Ca_ suggests the mechanism driving the reduced systolic calcium transient is impaired SR calcium cycling. Indeed, we show a clear impact of SR inhibition on systolic calcium transient amplitude and time course of decay as expected based on the reliance of excitation‐contraction coupling on SR calcium cycling in sheep ventricle (Hutchings et al., 2022). Phenanthrene exposure did not further reduce the amplitude of the transient or further slow the decay suggesting phenanthrene and the SR inhibitors could be acting through the same pathways. SERCA is the primary mediator of calcium transient decay in sheep (Milani‐Nejad & Janssen, 2014), and prolongation in the decay rate (tau) observed here indicates that phenanthrene can impair calcium extrusion from the cytosol. Inhibition of SERCA mRNA (Atp2a2a gene) expression and protein levels have been previously observed in both zebrafish and rat embryonic cardiac myoblasts following 72 h of phenanthrene exposure (Zhang et al., 2013). In rainbow trout ventricular homogenates, phenanthrene (and other low molecular weight PAHs) were found to increase here SERCA activity in a dose‐dependent manner when applied acutely (Haverinen et al., 2024) which contrasts with our findings from intact cells here and in other studies (Ainerua et al., 2020; Brette et al., 2017). The same paper reports a decrease in SERCA expression levels when applied chronically (Haverinen et al., 2024).

A phenanthrene‐dependent change in SERCA activity could alter SR calcium content, causing the reduced calcium transient amplitude. Alternatively, the reduction in the amplitude may be due to altered calcium release from the SR through the SR calcium release channels/ryanodine receptors. Whether this is a direct effect of the PAH on the ryanodine receptor, or a result of a reduction in calcium‐induced calcium release following the marginal (~10%) reduction in the I_Ca_ trigger is unknown and awaits further work for clarification.

## 5 CONCLUSION

This is the first large animal study of the acute cardiotoxicity of phenanthrene, a ubiquitous PAH air pollutant present in both the gas‐phase and bound to PM. We show significant inhibition of I_Kr_, indicating ERG channel block at environmentally and physiologically relevant concentrations. However, the minimal impact on APD suggests a lower proarrhythmic risk linked to repolarization delay than that seen in fish (Marris et al., 2020) and small mammals (Yaar et al., 2023). We used a low (0.5 Hz) stimulation frequency to be able to clearly observe phenanthrene effects in paired experiments in our current study. However, it would be beneficial for future work to be conducted across a wider range of physiologically relevant frequencies to better understand proarrhythmic risk in this large animal model. The reduction in calcium transient amplitude across all levels of exposure used in this study (3, 10, and 25 μM) suggests a high risk of contractile dysfunction in large mammals following exposure. In fish (brown trout (Ainerua et al., 2020) and navaga cod (Filatova et al., 2024)), changes in calcium cycling following PAH exposure have been supported by changes in myocardial force production and pressure development, respectively. Thus, although changes in contractility need to be established, the findings predict contractile dysfunction during acute exposure in large mammals. Finally, while not investigated here, air pollution is known to increase ROS and induce mitochondrial dysfunction (Cai et al., 2019) which can impact excitation‐contraction coupling indirectly by changing the metabolic state of the cardiac myocytes. Thus, future work should directly investigate myocyte metabolism following phenanthrene exposure.

## AUTHOR CONTRIBUTIONS

CRM aided in animal husbandry, isolated sheep cardiomyocytes, conducted the calcium transient and membrane potential study, performed the corresponding data analysis, prepared figures, and drafted the manuscript. SNK conducted the patch‐clamp experiment, performed the corresponding data analysis, and edited the manuscript. AWT maintained the sheep colony and edited the manuscript. JCH and HAS conceptualized the study and were responsible for supervision, funding, and reviewing and editing the final version of the manuscript.

## FUNDING INFORMATION

This work was supported by British Heart Foundation grant PG/17/77/33125 to HAS and JCH and a University of Manchester PhD scholarship to CRM.

## CONFLICT OF INTEREST STATEMENT

The authors declare no conflicts of interest.

## ETHICS STATEMENT

All procedures involving the use of animals were performed in accordance with The United Kingdom (UK) Animals (Scientific Procedures) Act, 1986 and European Union Directive 2010/63. Local ethical approval was obtained from the University of Manchester Animal Welfare and Ethical Review Board.

## Data Availability

All data will is available to download from figshare https://doi.org/10.6084/m9.figshare.29613599.
